# Prehospital risk stratification following out-of-hospital cardiac arrest

**DOI:** 10.1186/cc12235

**Published:** 2013-03-19

**Authors:** Y Goto, T Maeda, Y Goto

**Affiliations:** 1 Kanazawa University Hospital, Kanazawa, Japan; 2 Yawata Medical Center, Komatsu, Japan

## Introduction

Multivariate analyses have identified factors that have enabled the development of sophisticated equations and scoring models with the ability to predict outcomes following out-of-hospital cardiac arrest (OHCA). However, implementation of such outcome prediction models in research and clinical practice has been slow. The more crucial aspect of these predictions is the lack of prehospital risk stratification for OHCA patients. Prehospital risk stratification for patients after OHCA may help clinicians guide in-hospital strategies, particularly in the emergency department. The purpose of this study was to develop a simple and generally applicable prehospital risk-stratification scheme for patients after OHCA.

## Methods

We analyzed data for 390,226 adult patients (age ≥18 years) after nontraumatic OHCA, from a prospectively recorded nationwide Utstein-style Japanese database for 2005 to 2009. The endpoint was 1-month survival with favorable neurological outcome (Cerebral Performance Category (CPC) 1 to 2).

## Results

Multivariate logistic regression analysis indicated the following three prehospital variables as significant high-ranking factors for predicting favorable 1-month outcomes: shockable initial rhythm (odds ratio (OR), 5.87; 95% CI, 5.23 to 6.60), witnessed arrest (OR, 3.05; 95% CI, 2.83 to 3.28), and age (≥18 to <71 years; OR, 3.24; 95% CI, 2.97 to 3.53). Using recursive partitioning analysis for development cohort data (2005 to 2008, *n *= 307,896), we stratified prehospital risk: if OHCA with shockable initial rhythm was witnessed, the probability of CPC 1 to 2 was 20.0% (age, ≥18 to <71 years; grade 1) or 10.3% (age, ≥71 years; grade 2); if OHCA with shockable initial rhythm was unwitnessed, the probability was 6.8% (age, <81 years; grade 3) or 1.8% (age, ≥81 years; grade 4a); if OHCA with unshakable initial rhythm was witnessed, the probability was 1.4% (grade 4b); and if OHCA with unshakable initial rhythm was unwitnessed, the probability was 0.3% (grade 5). The *c*-statistics for risk stratification of the development and validation cohorts (2009 external data, *n *= 82,330) were 0.853 (95% CI, 0.846 to 0.859) and 0.875 (95% CI, 0.865 to 0.885), respectively. The odds ratios for CPC 1 to 2 at 1 month between patients at very high and very low were 79.5 (95% CI, 72.5 to 87.4) and 122.1 (95% CI, 102.0 to 147.3), respectively, in these cohorts.

## Conclusion

Prehospital risk stratification (grade 1 to 5) of patients after OHCA using three prehospital factors (shockable initial rhythm, witnessed arrest, and age) accurately classifies the severity of OHCA and may help clinicians guide in-hospital strategies.

